# Aspect-Aided Dynamic Non-Negative Sparse Representation-Based Microwave Image Classification

**DOI:** 10.3390/s16091413

**Published:** 2016-09-02

**Authors:** Xinzheng Zhang, Qiuyue Yang, Miaomiao Liu, Yunjian Jia, Shujun Liu, Guojun Li

**Affiliations:** 1 College of Communication Engineering, Chongqing University, Chongqing 400044, China; 20151213053@cqu.edu.cn (Q.Y.); 20151202018@cqu.edu.cn (M.L.); yunjian@cqu.edu.cn (Y.J.); liusj@cqu.edu.cn (S.L.); 2 Department of Communication Commanding, Chongqing Communication Institute, Chongqing 400035, China; ly007@cqu.edu.cn

**Keywords:** microwave imaging sensor, image classification, aspect angle, sparse representation

## Abstract

Classification of target microwave images is an important application in much areas such as security, surveillance, etc. With respect to the task of microwave image classification, a recognition algorithm based on aspect-aided dynamic non-negative least square (ADNNLS) sparse representation is proposed. Firstly, an aspect sector is determined, the center of which is the estimated aspect angle of the testing sample. The training samples in the aspect sector are divided into active atoms and inactive atoms by smooth self-representative learning. Secondly, for each testing sample, the corresponding active atoms are selected dynamically, thereby establishing dynamic dictionary. Thirdly, the testing sample is represented with $\ell_{1}$-regularized non-negative sparse representation under the corresponding dynamic dictionary. Finally, the class label of the testing sample is identified by use of the minimum reconstruction error. Verification of the proposed algorithm was conducted using the Moving and Stationary Target Acquisition and Recognition (MSTAR) database which was acquired by synthetic aperture radar. Experiment results validated that the proposed approach was able to capture the local aspect characteristics of microwave images effectively, thereby improving the classification performance.

## 1. Introduction

**Figure 1:**
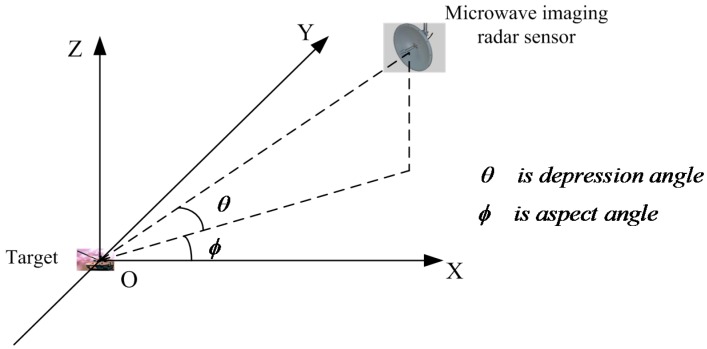
Description of aspect angle and depression angle in microwave imaging radar sensor.

**Figure 2:**
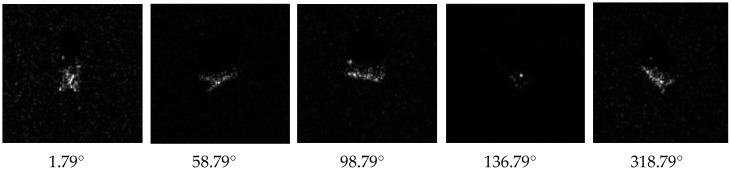
Microwave images of BMP2 at different aspect angles (depression angle 17°).

Microwave imaging sensors have gained extensive applications in bio-tissue image sensing, concealed weapon detection, and aerial remote sensing [1,2,3], in which the first and foremost objective is target microwave image classification. The present study is focused on target classification in images collected by a microwave imaging radar. Microwave imaging of targets is basically based on the electromagnetic scattering mechanism, so the images are usually not as detailed as optical images. Moreover, microwave imaging is very sensitive to the aspect angle and depression angle, so changes of these angles can cause significant changes in images. Figure 1 describes the aspect angle and depression angle in microwave imaging radar. In Figure 2, the images of a BMP2 target at varying aspect angles are given with a constant depression angle of 17°. As shown, image information differed profoundly for different aspect angles. Thus, these factors increase the difficulty in microwave image recognition [4,5]. 

There are two primary types of target classification technology used for images of microwave imaging radar: the template-based method [6] and model-based method [7]. The principle underlying the template-based method involves extracting features and comparing them with the stored templates generated by the training images, thereby identifying the unknown test image as the class of the matched template. The problem is that background clutters in microwave images interfere profoundly with template matching. The sensitivity to aspect angle and depression angle also causes difficulties in template matching. For the model-based method, SAR images were described using a statistical model based on scattering mechanisms. Then the target’s type was determined based on the maximum posterior probability of model parameters. Because of the difficulty of parameter estimation, this process can easily produce in weak correlation between training and testing samples, causing the method to fail. In addition, these methods have much worse classification performance at extended operating conditions, because the working parameters of training samples and testing samples are significantly different. Other optional methods include discriminative tensor analysis, non-negative matrix factorization (NMF), etc. [8,9].

In recent years, the sparse representation theory has been widely adopted in image processing and pattern recognition. The principle is to represent observed signal using linear combinations of a series of known signals called atoms. In sparse constraint of representation coefficient (minimal $\ell_{0}$ norm), a unique solution was obtained. Then the target type was determined by minimizing the reconstruction error of every type of training sample. Sparse representation has been used extensively in various recognition tasks, such as facial recognition, speech recognition, and hyperspectral image classification [10,11,12]. Sparse representation based recognition has gained in-depth development in accordance to different requirements of image recognition. Fang et al. investigated multi-task sparse learning algorithm [13]. In a previous study by Zhang et al., the kernel technique and sparse representation in non-linear processing were used to create a kernel sparse representation model, which was then used in facial recognition and hyperspectral image classification [14]. Meanwhile, the $\ell_{1}$-regularized non-negative least square (NNLS) sparse representation was employed in classification of gene data [15,16]. The NNLS sparse representation model possessed advantages of clear physical meaning, high classification accuracy and rapid calculation.

In microwave radar image recognition, several studies have focused on sparse representation- based classification. For example, Zhang et al. proposed a joint sparse representation target classification method by evaluating the correlation between multi-view microwave images [17]. On this basis, Chen et al. presented an improved joint sparse representation by using low-rank matrix recovery [18]. Dong et al. studied monogenic signal joint sparse representation-based target recognition of microwave image sensors, and further developed sparse representation of monogenic signals on Grassmann manifolds [19,20]. Liu et al. investigated microwave image recognition via multiple sparse Dempster-Shafer fusion [21]. Liu et al. introduced fusion of sparse representation and support vector machine for microwave radar image target recognition [22]. Cao et al. proposed a target classification method based on joint sparse representation with multi-view SAR images over a locally adaptive dictionary [23]. Hou et al. investigated SAR image classification with hierarchical sparse representation and multisize patch features [24]. Recently, there are also several techniques based on sparse representation for polarimetric SAR image classification [25,26,27,28]. 

In current microwave image recognition methods involving sparse representation theory, training samples of all aspect angle from 0° to 360° were considered as dictionary atoms. It is assumed that the all aspect angle microwave images of one type of target exist in a linear subspace. In the presence of large differences in aspect angle, the assumption is inappropriate due to the sensitivity of microwave images to aspect angle. According to the scattering characteristics of a target, the microwave images are, in essence, characterized by the electromagnetic scattering mechanism. According to this, only microwave images with approximately the same aspect angles have strong correlation, and those with different aspect angles have weak correlation. This is in fact the aspect-dependent structural characteristics of microwave images. For example, images at 6° aspect angle have a close correlation with images with 1°–11° aspect angle, and the correlation with other aspect angle images is weak, especially when the difference in aspect angle is large. In this way, the sparse representation of a testing image should only involve training samples with high aspect correlation with the testing image, rather than all SAR training images. Meanwhile, the dictionary matrix is not optimal if constituted by all of 0°–360° training samples. The production of excessive numbers of dictionary entries might render computation and storage unnecessarily taxing.

**Figure 3:**
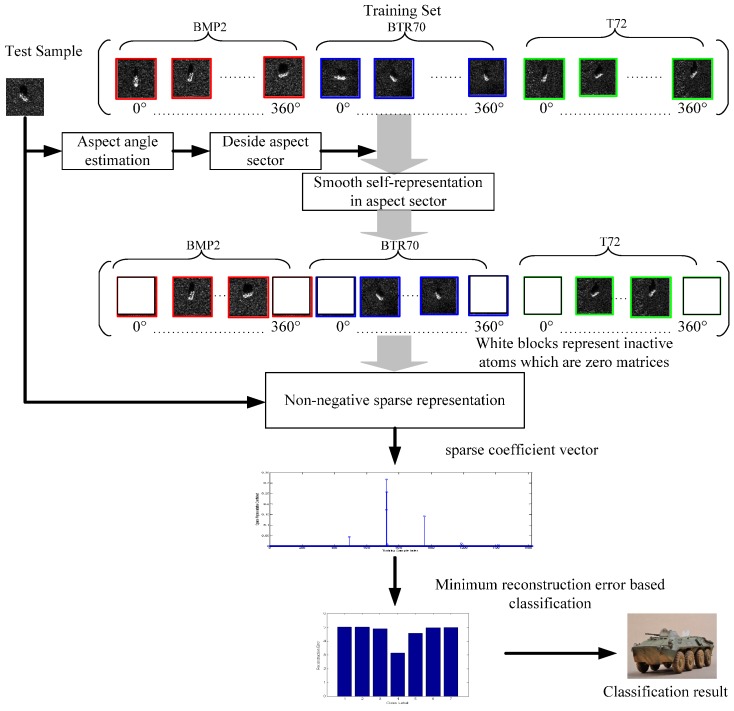
Flowchart of microwave image recognition based on aspect-aided dynamic non-negative least square sparse representation.

The main contribution of the current study lies in the presentation of a microwave image recognition method based on aspect aided dynamic non-negative least square (ADNNLS) sparse representation. Figure 3 shows a schematic view of the proposed algorithm. First, the aspect angle of a testing sample was estimated. Taking the estimated aspect angle as the center, a sector was then defined. Training samples of all targets in the sector were considered effective dictionary atoms, others not in the sector were null atoms, and replaced with zero matrices. However, estimations of aspect angle are subject to errors. To prevent actual dictionary atoms not falling into an effective sector, the sector cannot be too small, so there are still some atoms in the sector that have weak correlations with the testing sample. In order to further narrow down the range of effective atoms, they were divided into two groups via smooth self-presentation clustering learning. The first group considered the testing sample to be the clustering center, and the second considered the furthest training sample to the testing sample to be the center. It is worth noting that the clustering samples included the testing sample, rather than only training samples in the sector. In this way, the selection of effective atoms could be enhanced. Then, only the effective atoms in the testing sample group were preserved. These are here called active atoms. The remaining atoms were set to zero matrices. Hence, the corresponding dictionary atoms of different testing samples need to be updated dynamically. That is why it is called aspect-dependent dynamic sparse representation. In this way, the size of dictionary did not change, with the active atoms keeping their original value and invalid atoms becoming zero matrices. Then the $\ell_{1}$-regularized non-negative least square sparse representation was used to perform regression representation of the tested sample. Decision making of the testing sample was then performed based on the principle of minimum reconstruction error. The advantage of the proposed model is that the quantity of effective data in training samples was reduced, and the interference of invalid atoms with large differences in aspect angle was eliminated, thereby improving the recognition accuracy. Additionally, the large number of zero matrices in the dynamic dictionary helps to reduce the required computation time and data storage space in sparse solving. Based on the Moving and Stationary Target Acquisition and Recognition (MSTAR) public database, several experiments were conducted to evaluate the performance of the proposed algorithm. The target microwave images in the database covered 0°–360° aspect angles, which enabled algorithm verification of the proposed aspect-aided dynamic non-negative least square sparse representation. The results were then compared to those of state-of-art algorithms.

This paper is organized as follows: Section 2 describes the algorithm for aspect-aided dynamic selection of active atoms. In Section 3, the non-negative least square sparse representation algorithm is reviewed. Then the microwave image recognition algorithm based on aspect-aided dynamic non-negative least square sparse representation is presented. In Section 4, experiments carried out according to the MSTAR database and comparisons between the proposed algorithm and the current algorithm are described. Conclusions are given in Section 5.

## 2. Aspect-Aided Dynamic Active Atoms Selection

The characteristics of target microwave images obtained from microwave imaging radar are closely related to their aspect angle. When the aspect angle changes severely, the pixel distribution in the target images also show abrupt changes. In this study, only the influence of the aspect angle was analyzed because it had the most dramatic influence on microwave images characteristics, and the depression angle did not change much in the experimental data.

### 2.1. Estimation of the Testing Sample Aspect Angle

As given above, the aspect angle of a testing sample needs to be estimated in the microwave image recognition algorithm based on ADNNLS sparse representation. It was also needed in template matching microwave image recognition [6]. Current estimation methods, which are only based on the information of the testing sample images, mainly use a technique such as radon transformation to estimate the target aspect angle [29]. The current methods did not consider using training samples with known aspect angles. Actually, in microwave image recognition tasks, there are large numbers of training samples with known aspect angle, which can be used to estimate the testing sample aspect angle. Thus, we present a simple but effective testing sample aspect angle estimation method based on the training sample aspect angle. This is an innovation of the current study. The precondition of the method is that the training sample aspect angle is known and distributed evenly across 0°–360°. The principle of the method is that the testing sample is closely correlated to training samples which have similar aspect angle. Hence, the correlation coefficients between the testing samples and training samples were calculated, and the aspect angle of training samples with the largest correlation coefficient was taken as the estimation value of the testing sample. Assuming that the testing sample are *A*, and certain training sample is *B*, the correlation coefficient *r* could be calculated as below:

(1) $$r=\frac{\sum_{m}\sum_{n}\left(A_{mn}-\bar{A})(B_{mn}-\bar{B}\right)}{\sqrt{\left(\sum_{m}\sum_{n}{\left(A_{mn}-\bar{A}\right)}^{2}\right)\left(\sum_{m}\sum_{n}{\left(B_{mn}-\bar{B}\right)}^{2}\right)}}$$

Herein, $\bar{A}$ represents the mean of ***A***, $\bar{B}$ is the mean of ***B***, and ***A***_*mn*_, ***B****_mn_* represent the value of pixels at row *m* and column *n* in ***A*** and ***B***, respectively.

**Figure 4:**
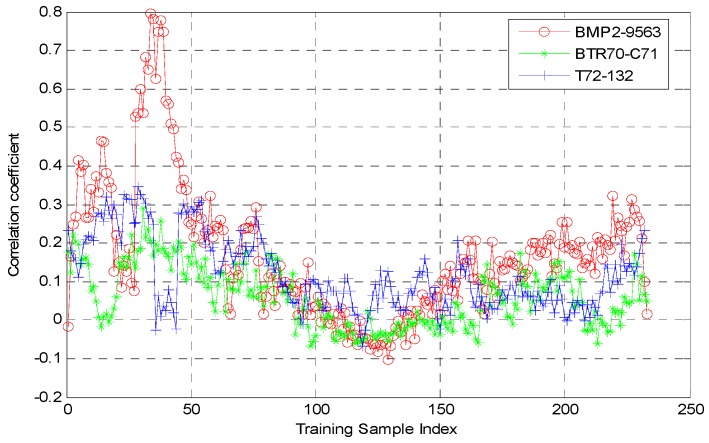
Correlation coefficient curve between BMP2-9563 testing sample (depression angle 15°, aspect angle 48.49°) and all training samples of targets BMP2-9563, BTR70-C71, and T72-132.

**Figure 5:**
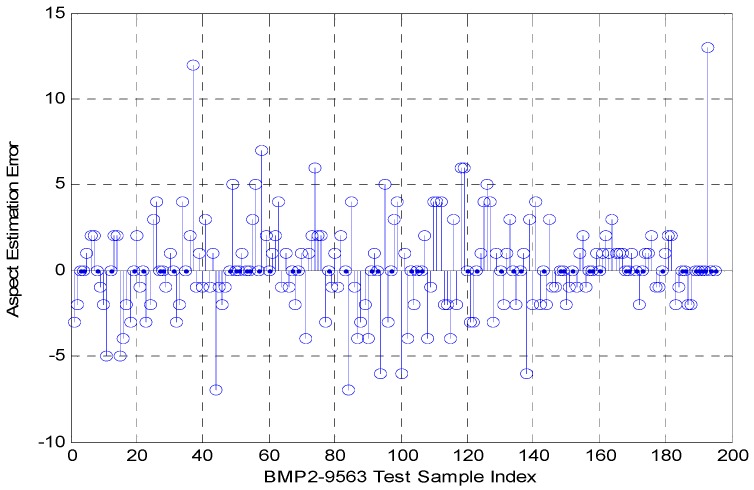
Estimation error in aspect angle of all testing samples of BMP2-9563.

The microwave images of target BMP2-9563 were used for verification. Correlation analyses were performed between one testing image of target BMP2-9563 at 15° depression angle and 48.49° aspect angle and training images of targets BMP2-9563, BTR70-C71, and T72-132 at 17° depression angle and all aspect angles. Figure 4 shows the variation curve of the correlation coefficients. As shown, the training samples with largest correlation coefficient were the 34th training samples of BMP2-9563. The corresponding aspect angle was 46.49°, which was then taken as the estimation value of testing sample aspect angle. As shown, there was an error of 2° relative to the true aspect angle of the testing sample. Figure 5 shows the errors of all testing samples in BMP2-9563. As shown, the error was around 5° for most cases and slightly above 10° for some. Data analysis showed that the estimated aspect angle mean error for BMP2-9563 testing samples was 0.19°, and the standard deviation is 2.76°. A similar trend was found for other testing samples in experiments.

This method of estimation has the following advantages: (1) it fully utilizes the aspect angle information of the training samples; (2) the calculations were simple; and (3) the problem of distinguishing target head and tail while only using testing samples information was avoided.

Once the aspect angle of the testing sample has been estimated, it was taken as the center to determine a sector. For example, if the testing sample aspect angle estimated is 40°, we can determine a sector which aspect angle range is from 25° to 55°. All types of training sample subsets in the sector were considered effective atoms. The other training samples not in the sector were set to zero matrices. A preliminary dictionary was composed of the effective atoms and zero matrices atoms.

### 2.2. Enhanced Atoms Selection in Aspect Sector Based on Smooth Self-Representation 

The corresponding aspect sector was determined based on aspect angle estimation of testing sample, and the effective atoms were chosen. However, as shown above, the maximum estimation error might be as high as 15°. In order to prevent omission of any effective atoms, the sector size should be at least ±15°, namely covering an area of 30°. Hence, the effective atoms subsets contained redundancy. The smooth self-representation learning clustering method was adopted in this study to further refine these subsets. The clustering reported by Shafiee et al. was only for the training samples themselves [30]. Here, clustering was performed for both the testing sample and training samples. The aim of clustering was to divide effective atoms into two groups, one group containing the atoms closely correlated to the testing sample, and the other group had low correlations to the testing sample. Therefore, clustering was conducted with two centers, the current testing sample and the atom furthest from the current testing sample.

The smooth self-representation clustering algorithm presented by Han et al. was used in this study [31]. In clustering, the grouping effect is very important. The enforced grouping effect conditions are introduced in the smooth self-representation algorithm. This is what allows the algorithm to achieve such a strong clustering effect.

Assuming the dataset of the testing sample and effective training atoms is $X=[x_{1},x_{2},\cdots x_{n}]\in {\mathbb{R}}^{m\times n}$ where ***x****_i_*, *i* = 1,…,*n* is a vector with every sample arranged in columns. *x*_1_ is the testing sample, *x_n_* is the atom farthest from the testing sample. The clustering process is as below [31]:

First, the number of subspaces from clustering was obtained as *d* = 2, with two clustering centers *x*_1_ and *x_n_*. Then a K-NN plot ***W*** was made, and the corresponding Laplacian matrix $\tilde{L}$ was calculated [31].

It is necessary to calculate the self-representation matrix *U*, i.e., the following optimization problem:

(2) $$\underset{U}{\min}\;f(U)=\alpha {\left\|X-XU\right\|}_{F}^{2}+\mathrm{tr}(U\tilde{L}U^{T})$$

Here, α>0 is a control parameter, $\|\cdot {\|}_{F}$ represents the matrix norm, and tr(·) is the trace of the matrix. Equation (2) is a smooth convex programming problem. According to [31], the optimal solution U* could be found by solving the Sylvester matrix in Equation (3):

(3) $$\alpha X^{T}XU^{*}+U^{*}\tilde{L}=\alpha X^{T}X$$

Thereafter, the affinity matrix ***J*** was calculated using Equation (4):

(4) $$J=\left(\left|U^{*}\right|+\left|U^{*T}\right|\right)/2$$

Then, the final clustering result was obtained via the spectral clustering algorithm [31,32].

According to the clustering results, the atoms in the testing sample-centered type were refined effective atoms, here called active atoms. The other type of atom was replaced with zero vectors. Thus, the active atoms and zero vectors atoms constituted the final dictionary.

## 3. Non-Negative Least Square Sparse Representation Classifier

### 3.1. Basic Sparse Representation Classifier

Lately, sparse representation has gained widespread applications in image restoration, pattern recognition and hyperspectral image classification [33]. The success of the sparse signal model mainly lies in sparse representation of observed signal with a group of known observation samples (dictionary). The optimal sparse representation could be solved via the convex optimization method [34,35].

Given sufficient *k* types of training samples (*k* = 1,…,*K*), and $X_{k}=\left[x_{k,1},x_{k,2},\ldots ,x_{k,n_{k}}\right]\in {\mathbb{R}}^{m\times n_{k}}$, the samples were sorted in a column vector in space ${\mathbb{R}}^{m}$. An unknown observation sample $y\in {\mathbb{R}}^{m\times n_{k}}$ was represented as the linear combination of the *k-*th type training samples with the representation coefficient $\alpha_{k}={\left[\alpha_{k,1},\alpha_{k,2},\ldots ,\alpha_{k,n_{k}}\right]}^{T}\in {\mathbb{R}}^{n_{k}}$.

(5) $$y=x_{k,1}\alpha_{k,1}+x_{k,2}\alpha_{k,2}+\ldots +x_{k,n_{k}}\alpha_{k,n_{k}}$$

It was assumed that a certain type of sample formed an approximate linear subspace. Because the class label of observation sample ***y*** was unknown, it was represented with all *k* types of training samples of the training set. $X=\left[X_{1},X_{2},\ldots ,X_{K}\right]\in {\mathbb{R}}^{m\times n}$, here $n=\sum_{k=1}^{K}n_{k}$ is the total number of training samples. Then the linear representation of ***y*** was written as follows:

(6) $$y=X_{1}\alpha_{1}+X_{2}\alpha_{2}+\ldots +X_{K}\alpha_{K}=X\alpha$$

Herein, $\alpha ={\left[\alpha_{1},\alpha_{2},\ldots ,\alpha_{K}\right]}^{T}\in {\mathbb{R}}^{n}$ where ${\mathbb{R}}^{n}$ is the representation coefficient vector. Theoretically, the optimal representation *α* is a coefficient vector, in which the elements corresponding to observation samples *y* were non-zero, and the other elements were all zero.

When *m < n*, the solution of Equation (6) is not unique. A common method was to add sparse constraint term to the representation coefficient vector, thereby searching for the sparsest representation as below [36]:

(7) $$\underset{\alpha }{\min}{\left\|\alpha \right\|}_{0}\;s.t.{\left\|y-X\alpha \right\|}_{2}\leq \varepsilon$$

Herein, $\|\cdot {\|}_{0}:$ represents the $\ell_{0}$-norm of ***α***, and *ε* is the allowable error. Since the optimization problem of minimal$\;\ell_{0}$-norm is NP-hard, Equation (7) could only be approximately solved with certain algorithms, such as matching pursuit and orthogonal matching pursuit [37,38]. According to the compressed sensing theory [39], Equation (7) could be relaxed to $\ell_{1}$-norm minimization as below.

(8) $$\underset{\alpha }{\min}{\left\|\alpha \right\|}_{1}\;s.t.{\left\|y-X\alpha \right\|}_{2}\leq \varepsilon$$

Herein, $∥\cdot {∥}_{1}$ is the $\ell_{1}$-norm of ***α***. Since Equation (8) is a convex optimization problem, it could be solved with a traditional optimization algorithm [40].

In sparse representation classifier, after the optimal sparse representation was obtained, the class label of ***y*** could be determined based on the law of minimum reconstruction error:

(9) $$identity(y)=\underset{k=1,\ldots ,K}{\min}{\left\|y-X_{k}\alpha \right\|}_{2}$$

The above is the common-used basic sparse representation classifier (SRC) [41].

### 3.2. Non-Negative Least Square Sparse Representation Classifier

In SRC, the sparse representation coefficient might be either positive or negative, although a negative value has no physical meaning in actual application. Because of the presence of negative coefficients, different atoms might be eliminated as a result of offset, which does not conform to the visual perception mechanism. Previous studies have suggested that non-negative property was more accordant with human visual perception mechanism, therefore providing more effective representation [15,16]. Meanwhile, to a certain extent, non-negativity caused further sparsity. In terms of microwave image recognition, the non-negative constraint can eliminate atoms in sparse solution that are negatively correlated to input images, which is more reasonable for microwave image recognition. In the present study, the $\ell_{1}-$egularized non-negative least square sparse representation classifier was adopted [16]:

(10) $$\underset{\beta }{\min}\frac{1}{2}{\left\|y-X\beta \right\|}_{2}^{2}+\lambda {\left\|\beta \right\|}_{1}\;s.t.\;\beta \geq 0$$

Herein, ***y*** is the testing sample, ***X*** is the dictionary of training samples, and *λ* is the control parameter. Equation (10) could be transformed into the following non-negative quadratic programming (NNQP) problem (11):

(11) $$\underset{\beta }{\min}\frac{1}{2}\beta^{T}H\beta \;+w^{T}\beta \;s.t.\;\beta \geq 0$$

Herein, $H=X^{T}X$ and $w=\lambda -X^{T}y$. The optimization problem (11) could be solved with the Active-Set algorithm [16].

After obtaining the non-negative sparse coefficient vector ***β***, the law of minimal reconstruction error was used to identify the label of the testing sample:

(12) $$identity(y)=\underset{k=1,\ldots ,K}{\min}{\left\|y-X_{k}\beta \right\|}_{2}$$

### 3.3. Microwave Image Classification with ADNNLS Sparse Representation

In summary, the steps of microwave image recognition based on ADNNLS sparse representation are listed in Algorithm 1 as follows:
**Algorithm 1:** ADNNLS sparse representation for microwave image recognitionInput:$X_{m\times n}:$ All types of training samples$Y_{m\times p}$: All test samplesOutput: the identity of $Y_{m\times p}$Steps:1)A test sample ***y****_i_* was selected from $Y_{m\times p}$, and its correlation coefficients vector $r_{y_{i}}$ with all training samples were calculated. The aspect angle of the testing sample $\theta_{y_{i}}$ was estimated based on the aspect angle of training samples that showed the largest correlation coefficient.2)An aspect sector was determined based on the estimated aspect angle, and all types of training samples in the sector were included in a set of effective atoms. Based on smooth self-representation learning clustering, the set of active atoms $X_{y_{i}}^{\prime }$ was obtained.3)A dynamic dictionary $X_{y_{i}}^{\prime \prime }$ was created according to $X_{y_{i}}^{\prime }$.4)Using non-negative least square sparse representation algorithm, the testing sample yi was represented with the dynamic dictionary $X_{y_{i}}^{\prime \prime }$, thereby deriving the representation coefficient $\beta_{y_{i}}$.5)Class label of testing sample yi was obtained based on the law of minimal reconstruction error.6)If all testing samples were classified, go to step 7). Otherwise, go back to step 1);7)End

## 4. Experiments and Discussion

### 4.1. Description of the Dataset

**Figure 6:**
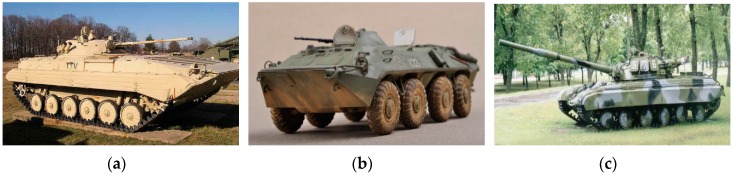
Optical images of three targets. (**a**) BMP2; (**b**) BTR70; (**c**) T72.

**Figure 7:**
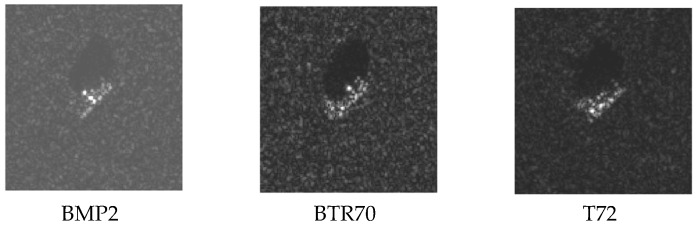
Microwave images of three targets at near aspect angles.

The MSTAR database of microwave images obtained using synthetic aperture radar was used in the experiments. The microwave imaging radar sensor worked in the X band, and the image resolution was 0.3 × 0.3 m. The images of three types of targets, i.e., BMP2, BTR70 and T72 were adopted. Optical images of the three targets are shown in Figure 6, and several microwave images of them with near aspect angle are shown in Figure 7. Specifically, the target BMP2 has three configuration types BMP2-9563, BMP2-9566, and BMP2-C21, BTR70 has one type BTR70-C71, and T72 has three configuration types T72-132, T72-812, and T72-S7. Table 1 lists the number of all training samples and testing samples. The microwave images at 17° depression angle of three targets were taken as training samples, and the images at 15° depression angle were testing samples. For each type of target, the images covered 0°–360° aspect angle. The sample type, depression angle, target type, and sample numbers are shown in Table 1. First, pre-processing was carried out for all training samples and testing samples by logarithm transformation to compress the dynamic range and to change multiplicative noise spots into additive noise. Because the original images contained background clutter, in order to reduce interference, a 60 × 60 m sub-image centered at the original image center was extracted, and all targets were located in the sub-image.

### 4.2. Classification Results 

**Figure 8:**
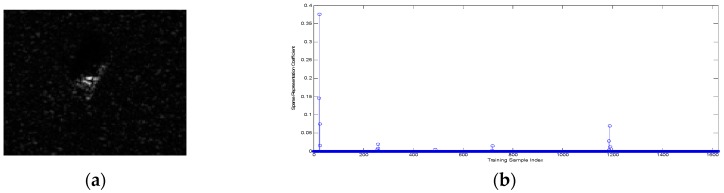

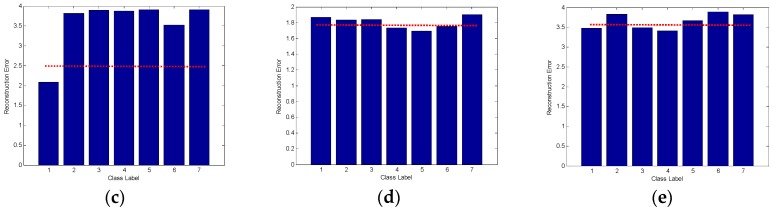
(**a**) Test sample BMP2-9563; (**b**) Sparse vector obtained based on ADNNLS algorithm; (**c**) Reconstruction error of ADNNLS algorithm; (**d**) Reconstruction error of SRC algorithm; (**e**) Reconstruction error of NNLS algorithm.

**Figure 9:**
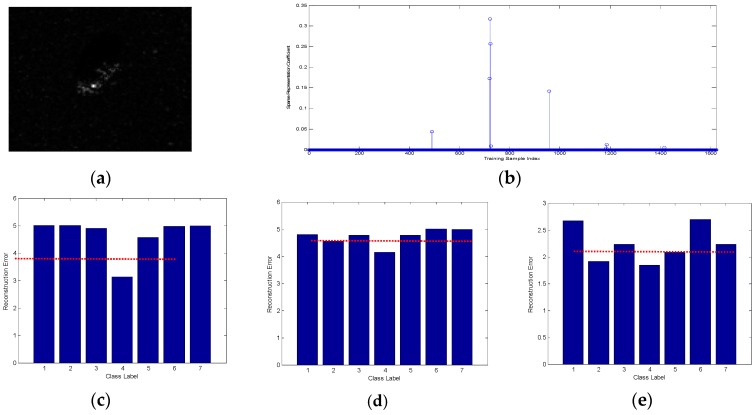
(**a**) Test sample BTR70-C71; (**b**) Sparse vector obtained based on ADNNLS algorithm; (**c**) Reconstruction error of ADNNLS algorithm; (**d**) Reconstruction error of SRC algorithm; (**e**) Reconstruction error of NNLS algorithm.

**Figure 10:**
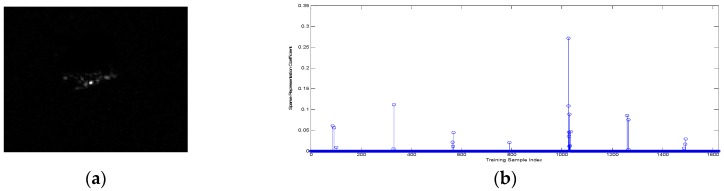

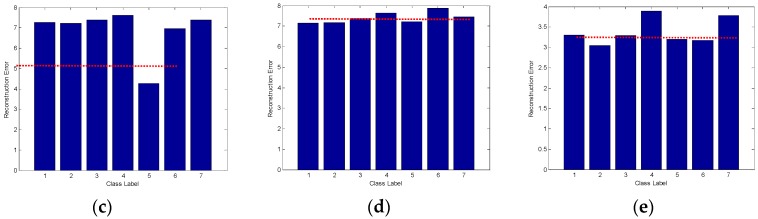
(**a**) Test sample T72-132; (**b**) Sparse vector obtained based on ADNNLS algorithm; (**c**) Reconstruction error of ADNNLS algorithm; (**d**) Reconstruction error of SRC algorithm; (**e**) Reconstruction error of NNLS algorithm.

The classification performance of the proposed ADNNLS algorithm was analyzed first. To address the advantages of ADNNLS in microwave images classification, one BMP2-9563 testing sample, one testing sample of BTR70-C71, and one testing sample of T72-132 were selected as the testing samples to assess the sparse coefficient distribution diagram and reconstruction error graph, which was then compared to that of SRC and NNLS. The dictionaries in SRC and NNLS comprised global aspect training samples of all target types, including 233 training samples of BMP2-9563, 232 training samples of BMP2-9566, 233 training samples of BMP2-C21, 233 training samples of BTR70-C71, 232 training samples of T72-132, 231 training samples of T72-812, and 228 training samples of T72-S7. In ADNNLS, the training samples were selected within the aspect sector after smooth self-representation clustering, so, even though the dictionary was the same size as SRC and NNLS, the number of effective non-zero atoms was much smaller than in the SRC and NNLS. Sparse coding was carried out for three testing samples with corresponding dictionary, thereby producing three sparse coefficient vectors and corresponding reconstruction error graphs, as shown in Figure 8, Figure 9 and Figure 10. As shown in Figure 8b, the maximum sparse coefficient corresponded to BMP2-9563. Comparison of Figure 8c–e suggested that minimal reconstruction error for SRC and NNLS corresponded to incorrect classification, while the minimal reconstruction error for ADNNLS was able to identify the correct type. As shown in Figure 9c–e, although three algorithms were able to correctly identify the type, the reconstruction error of BTR70-C71 sample in ADNNLS was far less than other types. Similarly, Figure 10 shows the classification of a testing sample of T72-132. Using SRC, it was incorrectly identified as BMP2-9563. When using NNLS, it was then identified as BMP2-9566. 

In traditional SRC and NNLS without the assistance of aspect angle, sparse decomposition was performed across global aspect range of 0°–360°, namely using training samples at all aspect angles. For the ADNNLS algorithm, the sparse decomposition and reconstruction were both performed within the local aspect sector, which was described in Algorithm 1 in detail. Therefore, because of the sensitivity of microwave images to aspect angle, ADNNLS was able to capture more aspect-related information compared with SRC and NNLS, not being interfered by training samples with large difference in aspect angle. These results indicated that the ADNNLS algorithm could prevent interference from training samples with large differences in aspect angle, thereby improving the classification performance.

**Figure 11:**
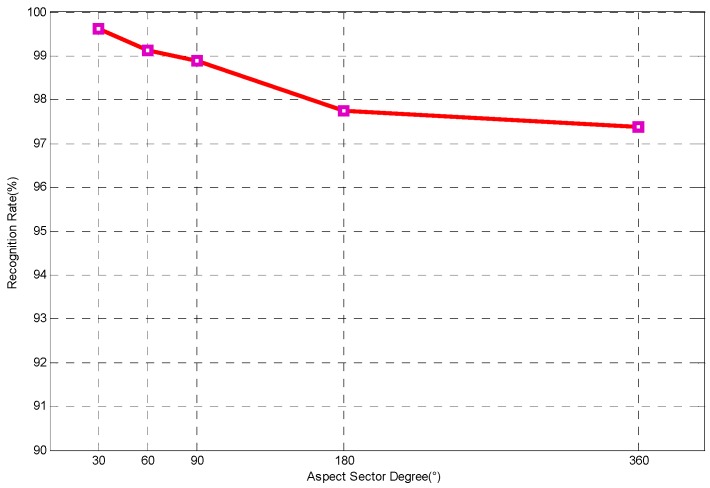
Variation in the performance of ADNNLS algorithm with the size of aspect sector.

In order to analyze the influence of sector size on ADNNLS performance, recognition experiments were carried out at different aspect sectors (30°, 60°, 90°, 180°, and 360°). Apparently, with 360° aspect sector, the algorithm was the full-aspect NNLS algorithm. Figure 11 shows the results of recognition. It can be seen that the recognition rate peaked at 99.63% for 30° aspect sector. As the sector increased, the rate gradually decreased, which was due to the increase of confusion atoms. Despite this, the recognition rate was still as high as 97.39% when the sector was 360°.

**Figure 12:**
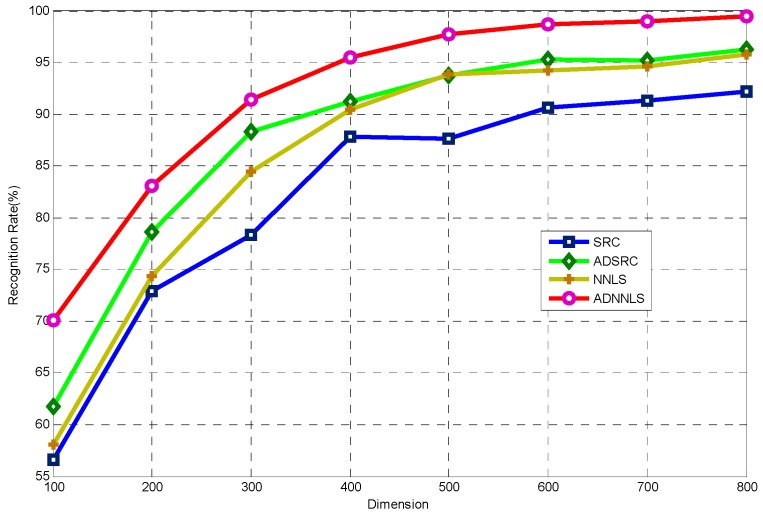
Recognition performance of the four algorithms with varying feature dimensions.

To assess the performance of the proposed algorithm, the recognition results of the current ADNNLS algorithm were compared to full-aspect SRC, aspect-aided dynamic SRC (ADSRC), full-aspect non-negative sparse representation (NNLS). A full-aspect algorithm means that the training samples across all aspects were included in the dictionary. An aspect-aided algorithm means the proposed aspect-aided smooth self-representation clustering was performed during dictionary design. The recognition experiments were conducted at different dimensions of PCA features. The results are as shown in Figure 12. 

The performance of each algorithm increased with increasing dimension. At 800 dimensions, ADNNLS showed the largest recognition rate, which was 99.49%. The maximal recognition rates for SRC, NNLS, and ADSRC were 92.23%, 96.8%, and 96.97%, respectively. Moreover, ADNNLS demonstrated good recognition performance at low dimensions. The results validated the effectiveness of the proposed algorithm in microwave images recognition. In addition, the results of the presented algorithm were compared to the results of recently published IJSRC algorithm [18]. The reason of choosing this article was that it used the same dataset as in the current study. The ADNNLS algorithm showed a recognition rate of 99.83% at 30° aspect sector, only two out of 1365 testing samples were incorrectly identified. For IJSRC, the recognition rate was 98.53%, less than our algorithm. IJSRC used multi-view joint sparse representation, which was different from ADNNLS.

**Figure 13:**
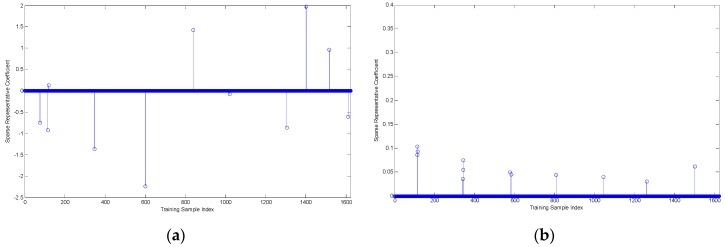

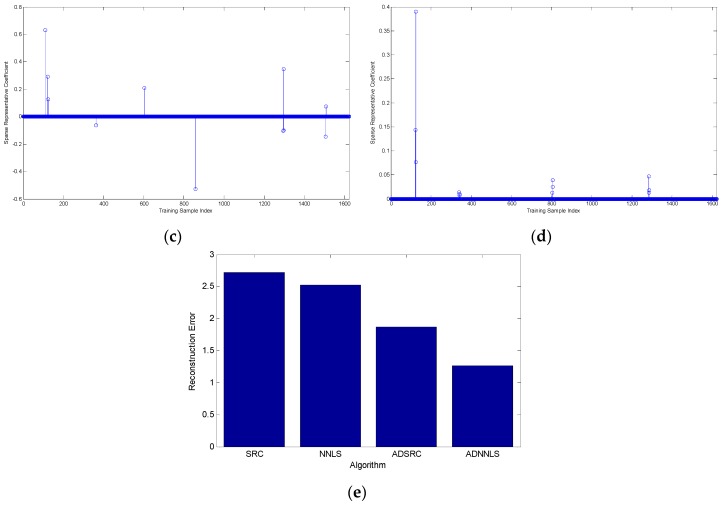
(**a**) Sparse coefficient vector obtained based on SRC algorithm; (**b**) Sparse coefficient vector obtained based on NNLS algorithm; (**c**) Sparse coefficient vector obtained based on ADSRC algorithm; (**d**) Sparse coefficient vector obtained based on ADNNLS algorithm; (**e**) Reconstruction error of four algorithms.

It is worth noting that the convergence condition used in four algorithms is error tolerance rather than sparsity in the manuscript. For comparing four algorithms performance with the same level of sparsity, we conducted the following experiment. We fixed sparsity value at 12, which means that non-zero elements number of solved sparse coefficient vector is 12. One query sample of BMP2-SN9563 with aspect angle 171.49° is selected to compare the representation coefficient vectors from the four algorithms. According to correspondence between training samples aspect angle and their index number, we know that samples near index number 114 should be selected and correspond to larger coefficient values. Figure 13 shows four representation vectors corresponding four algorithms. 

SRC and NNLS picked dispersed atoms without aspect angle information aided, which can be seen from Figure 8a,b. The atoms selected by ADSRC are more assembled than SRC and NNLS. However, from Figure 8d, it can be observed that ADNNLS can select most representative atoms near index number 114 corresponding larger coefficient values. Figure 8e shows that ADNNLS has minimum reconstruction error for this testing sample among four algorithms. The advantage of the ADNNLS is that it can reduce the interference of invalid atoms with large differences in aspect angle, thereby improving the recognition accuracy. 

**Figure 14:**
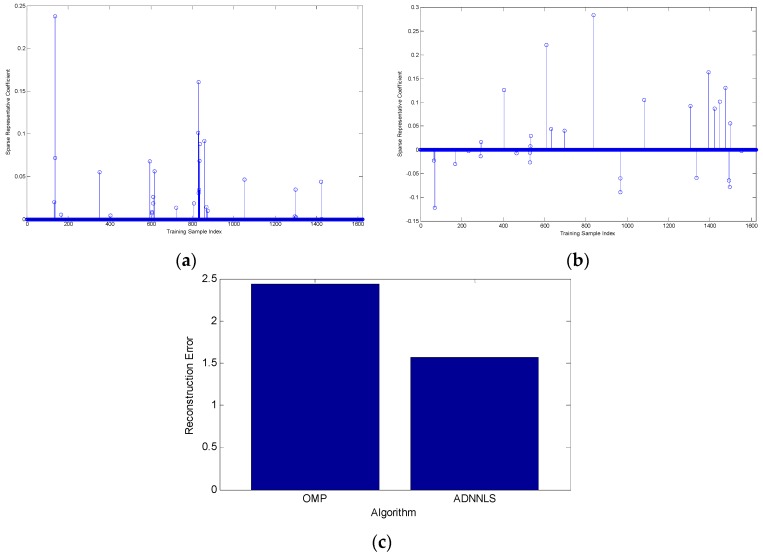
(**a**) Sparse coefficient vector obtained based on ADNNLS algorithm; (**b**) Sparse coefficient vector obtained based on OMP algorithm; (**c**) Reconstruction error of two algorithms.

We conducted another experiment to study whether OMP could pick similar dictionary atoms as ADNNLS by enforcing the same number of non-zero coefficients from ADNNLS. We select one BMP2-SN9563 testing sample with 213.49° aspect angle for this experiment. Ideally, those training samples near 213.49° in the dictionary will be picked under optimal representation algorithm. According to correspondence between training samples aspect angle and their index number, we know that samples near index number 135 should be selected and correspond to larger coefficient values. We first solve the representative coefficient vector under ADNNLS, which is shown in Figure 14a. The number of non-zero coefficients from ADNNLS is 29. The OMP representative vector is solved by enforcing 29 non-zero coefficients, which is shown in Figure 14b. From Figure 14a, it can be seen that there were four atoms near index number 135 picked and one atom corresponded to the largest value in the whole ADNNLS representative vector. However, there was only one atom near index number 135 selected with a small value under the OMP algorithm. From the OMP representing vector, we can see that atoms selected are more dispersed than those picked with ADNNLS. Although OMP is able to acquire a sparse solution, it cannot select those best represented atoms for a query sample which results in a larger representation error. Experiment results indicated that OMP had larger reconstruction error than ADNNLS, which can be seen from Figure 14c.

### 4.3. Robustess to Noise

**Figure 15:**
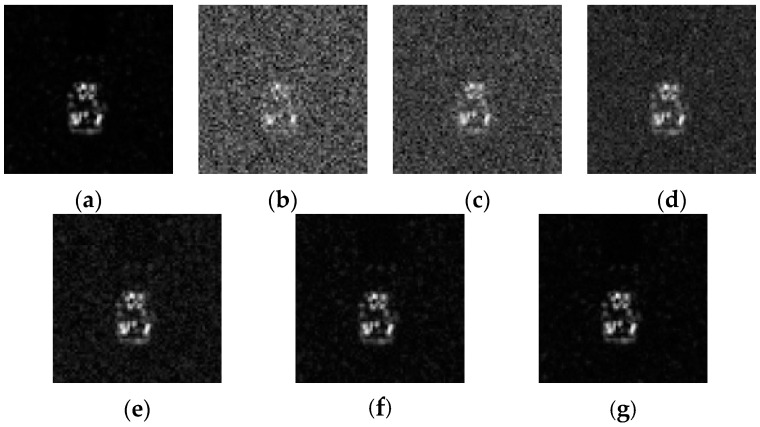
SAR images with different SNRs: (**a**) original image; (**b**) 0 dB; (**c**) 10 dB; (**d**) 20 dB; (**e**) 30 dB; (**f**) 40 dB; (**g**) 50 dB.

**Figure 16:**
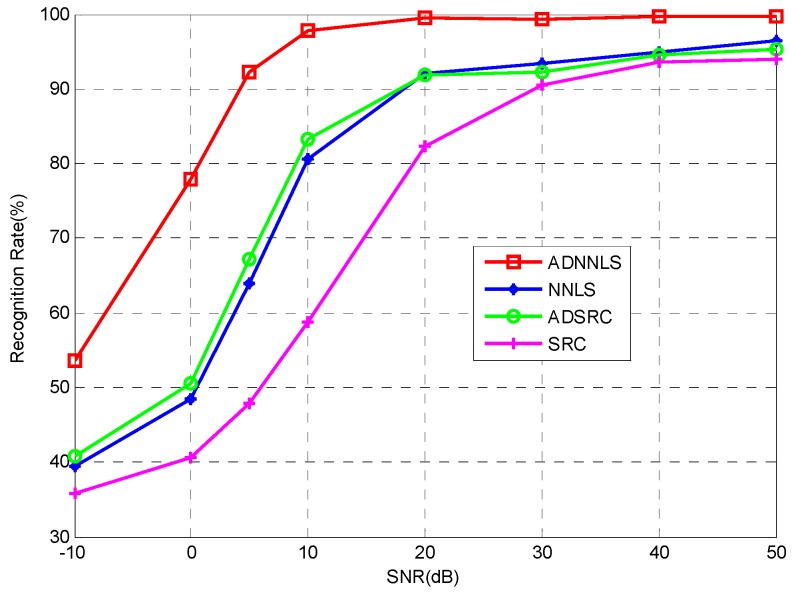
Performance of the algorithm with varying SNRs.

Additionally, the anti-noise performance of the proposed algorithm was investigated. Different level Gaussian noises with varying Signal-to-Noise Ratio values (SNRs) were added into the testing samples thereby evaluating the robustness of the algorithm. Figure 15 shows the SAR images at different SNRs. Figure 16 shows the recognition performance curves of four algorithms at different SNRs. From Figure 16, one can see that the recognition rate of all algorithms increase with higher SNR, however, the ADNNLS algorithm could reach a recognition rate of over 90% within a broad range of 5–50 dB, while, the other three algorithms all have recognition rates lower than 70% at 5 dB. The results show that ADNNLS is more robust to noise corruption than the other three algorithms.

### 4.4. Experiments Conducted with Depression Angle Variations

**Figure 17:**
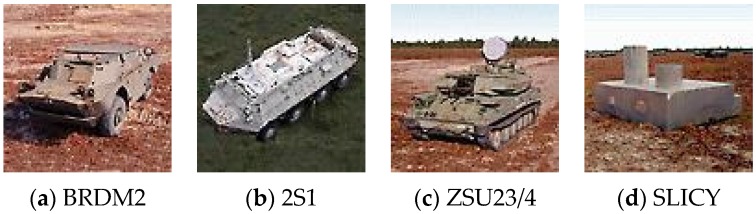
The optical images of four target. (**a**) BRDM2; (**b**) 2S1; (**c**) ZSU234; (**d**) SLICY.

**Figure 18:**
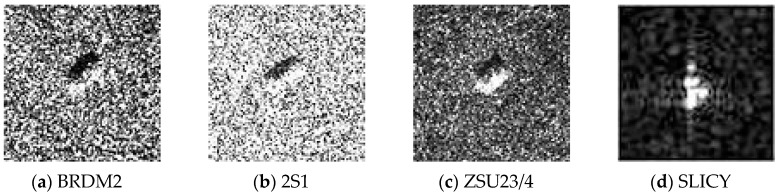
Example SAR images of four targets. (**a**) BRDM2; (**b**) 2S1; (**c**) ZSU234; (**d**) SLICY.

In addition to aspect angle changes, depression angle variations of test samples can also significantly affect recognition performance. In the experiments, four algorithms are also evaluated with a large depression angle variation. Four targets, BRDM2, 2S1, SLICY and ZSU23/4 are employed, as illustrated in Figure 17, several SAR image samples of these targets are illustrated in Figure 18. The dataset used in the depression variation experiments is shown in Table 2. In this experiment, images collected at 17° depression angle are used for training, while images taken at 30° and 45° are utilized for testing. Experimental results to be studied are shown in Table 3.

As can be seen from Table 3, with test samples at 30° depression angle, the accuracy of ADNNLS achieved a high level of 96.44%, 3.04%, 11.73% and 12.43% better in average than ADSRC, NNLS and SRC, but when test samples at 45° depression angle are used, the performance of the four algorithms all decline. The reason is that huge differences occur between the training and test samples when there are large changes of depression angle from 17° to 45°. In this condition, the over-fitting issue in all algorithms is much more serious. However, ADNNLS still achieves an accuracy of 79.21%, 1.4%, 14.11% and 19.39% better on average than ADSRC, NNLS and SRC.

The experimental results verify that ADNNLS is the most robust to the depression variation among the four algorithms. This is due to that aspect constraints and smooth self-representation that reduces the confusion caused by large depression variation, which ensures accurate inference ability of sparse representation. 

## 5. Conclusions

In the present study, an aspect-aided dynamic non-negative least square sparse representation model was proposed to classify target images collected by microwave imaging radar sensors, which fully utilized the aspect-dependent characteristic of microwave images. In ADNNLS sparse representation, the aspect sector of active atoms was determined based on the aspect angle of the testing samples. Then the smooth self-representation strategy was used in the sector to choose training samples that were most correlated to the testing sample, thereby constructing the dictionary. The strategy benefits searching for effective support training samples for the corresponding testing sample, and can effectively prevent interference from the training samples that have poor aspect correlation with the testing sample. Experiments based on MSTAR database showed that ADNNLS significantly outperformed SRC and NNLS and reached the recognition level of state-of-art algorithm. Meanwhile, it showed promising anti-noise behavior.

## Figures and Tables

**Table 1:** Name and number of the training and testing samples.

Number of Targets	1	2	3	4	5	6	7
Training sample type	BMP2	BMP2	BMP2	BTR70	T72	T72	T72
(17°)	sn-9563	sn-9566	sn-c21	sn-c71	sn-132	sn-812	sn-s7
Number	233	232	233	233	232	231	228
Testing sample type	BMP2	BMP2	BMP2	BTR70	T72	T72	T72
(15°)	sn-9563	sn-9566	sn-c21	sn-c71	sn-132	sn-812	sn-s7
Number	195	196	196	196	196	195	191

**Table 2:** Dataset used in the experiment.

	BRDM2	2S1	ZSU234	SLICY
**Training Set (17°)**	298	299	299	298
**Testing Set (30°)**	298	288	288	288
**Testing Set (45°)**	298	303	303	303

**Table 3:** Recognition rates under different depression angles with four classifiers.

Depression	Classifier			
	SRC	NNLS	ADSRC	ADNNLS
30°	84.41%	84.71%	93.40%	96.44%
45°	59.82%	65.10%	77.81%	79.21%
